# Efficacy of pyramiding elite alleles for dynamic development of plant height in common wheat

**DOI:** 10.1007/s11032-013-9873-5

**Published:** 2013-06-06

**Authors:** Bin Zhang, Wei Shi, Weiyu Li, Xiaoping Chang, Ruilian Jing

**Affiliations:** 1 National Key Facility for Crop Gene Resources and Genetic Improvement, Chinese Academy of Agricultural Sciences, Beijing, 100081 China; 2 Institute of Crop Science, Chinese Academy of Agricultural Sciences, Beijing, 100081 China

**Keywords:** Association mapping, Development, Elite allele, Gene pyramiding, Plant height, *Triticum aestivum*

## Abstract

Plant height is an important botanical feature closely related to yield. Two populations consisting of 118 and 262 accessions respectively were used to identify elite alleles for plant height and to validate their allelic effects. Plant height was measured from the early booting to the flowering stages. Simple sequence repeat markers for candidate quantitative trait locus (QTL) regions with large effects identified in a doubled haploid (DH) population (Hanxuan 10 × Lumai 14) were selected for further verification by association analysis. Nine loci significantly (*P* < 0.001) associated with plant height were detected 13 times in the population with 118 accessions. Three loci (*Xgwm11*-*1B*, *Xwmc349*-*4B* and *Xcfd23*-*4D*) were identified in three, two and two periods of plant height growth, respectively. Markers *Xbarc168*-*2D*, *Xgwm249*-*2D*, *Xwmc349*-*4B*, *Xcfd23*-*4D* and *Xgwm410*-*5A* located at or near additive QTL regions in the DH population proved to coincide with known *Rht* loci. The results showed a consistency between linkage analysis and association mapping, and also confirmed the value of fine mapping of QTL through combined linkage and association analyses. For final plant height, the alleles *Xgwm11*-*1B*_*208*_, *Xwmc349*-*4B*_*103*_ and *Xcfd23*-*4D*_*202*_ exhibited negative effects, i.e. reducing plant height; *Xwmc349*-*4B*_*101*_ and *Xcfd23*-*4D*_*205*_ showed significant positive effects. A second larger population (262 accessions) was used to validate the effects of these large-effect alleles and the efficacy of pyramiding in eight environments (year × site × water regime combinations). Strong correlations between final plant height and numbers of large-effect alleles indicated that the alleles contributed additively to plant height. The additive effects showed that pyramiding elite alleles for target traits has significant potential for wheat breeding.

**Electronic supplementary material:**

The online version of this article (doi:10.1007/s11032-013-9873-5) contains supplementary material, which is available to authorized users.

## Introduction

Plant height is an important botanical feature closely related to yield (Jiang et al. 2003). The use of dwarf and semi-dwarf wheat and rice varieties to increase crop yields was termed the “Green Revolution” (Hedden 2003). Most current leading cultivars in the northern China winter wheat region with heights of around 75–85 cm are even shorter than those with single semi-dwarfing genes (Zhou et al. 2007). However, the genetics of plant height are complex, with genes on 17 of the 21 wheat chromosomes having been reported (Börner et al. 1996). Twenty-two *Rht* genes with major effects have been described (McIntosh et al. 2008; Peng et al. 2011), but only *Rht*-*B1b* (formerly *Rht1*) and *Rht*-*D1b* (*Rht2*), carried by Norin 10, and *Rht8* are widely used in wheat breeding programs; varieties with one or more of these genes accounted for >70 % of current commercial wheat cultivars worldwide (Ellis et al. 2007; Hedden 2003). The wide use of dwarfing sources based on a limited number of key parents leads to relatively narrow genetic diversity, which reduces adaptation to various environmental conditions (Evenson and Gollin 2003; Reif et al. 2005; Roussel et al. 2004). Therefore, it is essential to determine the genetic basis of plant height and exploit elite alleles, i.e. the alleles with favorable effects for breeding high-yielding varieties.

The rapid development of molecular markers has provided a basis for detailed genetic analyses of complex traits, such as plant height, which involves several genes, and particularly for understanding interactions with environmental factors. Quantitative trait loci (QTL) associated with plant height were detected on almost all 21 chromosomes by linkage analysis and association mapping (Cadalen et al. 1998; Cui et al. 2011; Huang et al. 2006; Keller et al. 1999; Klahr et al. 2007; McCartney et al. 2005; Wang et al. 2010; Wu et al. 2010; Zhang et al. 2011). However, most of these studies measured only the final plant height, and did not annotate the quantitative variation on a time scale. Zhu proposed statistical methods for analyzing conditional genetic effects (Zhu 1995). By analyzing developmental behavior within the period (*t* − 1) to *t*, quantitative genetic effects can be revealed at specific stages excluding the effect of previous times (*t* − 1). Plant height is not only easily measured, but also undergoes obvious changes along with plant development. It is therefore a popular model trait for the study of quantitative gene expression in developmental genetics (Wu et al. 2010). Several studies on conditional genetic effects of plant height development are reported in wheat (Cui et al. 2011; Wang et al. 2010; Wu et al. 2010), rice (Cao et al. 2001; Yan et al. 1998a), soybean (Sun et al. 2006) and maize (Yan et al. 2003). The overall research shows that conditional QTL mapping is a valid way of revealing dynamic gene expression for height development, especially the epistatic effects (Cao et al. 2001). Using the conditional QTL mapping method, genetic relationships between plant height and plant height components have been evaluated (Cui et al. 2011). Plant height development is a network of genes expressed selectively during the whole period of plant height growth (Wu et al. 2010).

Molecular marker-assisted selection (MAS) is a breeding approach increasingly adopted to eliminate defects in elite breeding lines (Kuchel et al. 2007). Many QTL for plant height have been identified by linkage analysis, but two fundamental limitations restrict the use of marker-assisted crop improvement practices. Firstly, there must be validation of previously reported QTL across time, space and genetic background. Only adequately verified markers are useful for MAS (Wheeler et al. 2005). In addition, the better of two alleles identified by linkage analysis may not represent the best genotype in the potential germplasm pool available for breeding programs (Jestin et al. 2011). Furthermore, many factors influence QTL detection and the true genetic effects of a QTL are influenced by environment, year, population and sample size (Li et al. 2010). Linkage mapping using segregating populations often detects broad chromosome regions that result in low resolution due to the limited polymorphism between two parents. Thus, it is necessary to verify the robustness of reported QTL and to fine-map chromosome regions to detect more closely linked markers in order to identify elite alleles for MAS. In a previous study, 25 additive QTL for developmental behavior of plant height were detected in a doubled haploid (DH) population derived from the cross Hanxuan 10 × Lumai 14 (Wu et al. 2010).

In the current study, two populations were used, one for conditional and unconditional association mapping, and another for verifying large-effect alleles of associated loci in eight environments (year × site × water regime combinations). Plant height was measured from the early booting to the flowering stages and selection of simple sequence repeat (SSR) markers was based on candidate QTL detected in the previous research. The purpose was to (1) verify large-effect QTL previously detected in the DH population and further identify markers closely linked to plant height; (2) identify the large-effect alleles of associated loci; and (3) assess the efficacy of pyramiding large-effect alleles with two populations in nine environments.

## Materials and methods

### Plant materials

Two hexaploid winter wheat populations were used as the plant materials. Population 1 (118 entries) was used for conditional and unconditional association mapping of plant height. Population 2 (262 accessions) was used to validate the large-effect alleles of associated loci. All materials were sown at the beginning of October and harvested in the following mid-June. Each experimental unit was a two-row plot of 2 and 0.3 m between two rows, with 40 seeds planted per row.

Population 1 consisted of 50 accessions and 68 homozygous lines with uniform plant height derived from BC_2-3_F_2-5_ progenies of the accessions (Supplementary Table S1). The recurrent parents were Jinmai 47, Lumai 14 and Yumai 18. The accessions were planted in Changping, Beijing (116°13′E; 40°13′N) in 2009 (named environment E0). These materials were managed under natural rainfall conditions of 192 mm during the growing season.

Population 2 consisted of 262 accessions (Supplementary Table S2), of which 254 were from China, three from USA, two from Australia, two from Italy, and one from Romania, including 209 modern varieties, 43 advanced lines and 10 landraces (Li et al. 2012). The cultivars from China were mainly planted in the Northern Winter Wheat Zone, and Yellow and Huai River Valleys Facultative Wheat Zone over recent decades. The accessions were grown at two sites over 2 years, viz. Changping and Shunyi (116°56′E; 40°23′N) in Beijing, and the planting years were 2009 and 2010. At each site, the field was managed under two water regimes, rain-fed (DS) and well-watered (WW). The total rainfalls in the growing seasons were 192 mm and 131 mm, respectively. The WW plots were irrigated with 750 m^3^/ha at the pre-overwintering, jointing, flowering and grain filling stages. E1, E2, E3, E4, E5, E6, E7 and E8 indicate the environments of Changping in 2009 under DS, Changping in 2009 under WW, Shunyi in 2009 under DS, Shunyi in 2009 under WW, Changping in 2010 under DS, Changping in 2010 under WW, Shunyi in 2010 under DS, and Shunyi in 2010 under WW, respectively.

### Measurement of plant height at different growth stages

Plant height was measured every 7 days from the early booting stage until flowering, a total of four measurements, designated S_1_, S_2_, S_3_ and S_4_. S_4_ was the plant height at flowering, the final plant height. Based on development theory and QGAStation V1.0 software proposed by Zhu (Zhu 1995), conditional plant heights (${\text{S}}_{t} |{\text{S}}_{t - 1}$) were obtained from the data for unconditional plant height (S_1_–S_4_). For conditional plant heights, ${\text{S}}_{ 2} |{\text{S}}_{ 1}$ (PH_1_) revealed the net genetic effects of genes expressed during the first two stages (S_1_ and S_2_), and likewise for ${\text{S}}_{3} |{\text{S}}_{2}$ (PH_2_) and ${\text{S}}_{4} |{\text{S}}_{3}$ (PH_3_). All the phenotypic analyses were carried out using SAS V8.1 software.

### Genotype detection

Based on the result of our previous research on the Hanxuan 10 × Lumai 14 DH population, QTL with large effects on chromosomes 1B, 2D, 4B, 4D, 5A and 7B were selected as candidate QTL for further verification by association analysis (Wu et al. 2010). Twenty-nine candidate SSR markers covering the respective chromosome regions were used for association mapping (Supplementary Table S3). In addition, the population structure was evaluated by another 29 unlinked loci evenly distributed across the entire wheat genome (Supplementary Table S4). The genetic locations of all the SSR markers were obtained from the consensus map Ta-SSR-2004 (Somers et al. 2004). The fluorescent primers were synthesized by ABI (Applied Biosystems, Foster City, CA, USA). Amplification products were separated by an ABI3730 DNA Analyzer (Applied Biosystems), and the outputs were analyzed by GeneMapper software (http://www.appliedbiosystems.com.cn/). The allele number, allele frequency and polymorphism information content (PIC) were calculated by PowerMarker V3.25 software (Liu and Muse 2005). Marker alleles with frequencies of <5 % were treated as rare alleles.

### Population structure

Population structure was estimated by STRUCTURE v2.3.2 (Pritchard et al. 2000). The number of hypothetical subpopulations (*K*) was set from 2 to 9 with a burn-in period length of 50,000 iterations and a run of 500,000 replications of Markov Chain Monte Carlo (MCMC) after burn-in. Each *K* was duplicated five times. The admixture model of STRUCTURE allowed for a population mixture and correlated allele frequencies. The most appropriate *K* value was evaluated by ln*P*(*D*) in the STRUCTURE output (Evanno et al. 2005). According to the most appropriate *K* value, the Q-matrix of five repeats was integrated by using the CLUMPP software (Jakobsson and Rosenberg 2007).

### Association mapping

For marker-trait association, a structured association approach was implemented by a general linear model (GLM) in TASSEL 2.1 (Bradbury et al. 2007). In order to correct for spurious associations, the Q-matrix was used in the model. The threshold (*P* value) for significant association between markers and traits was 0.001. The phenotypic variance explained (PVE) for each significantly associated locus was evaluated by *R* ^2^ values for the markers (Zhang et al. 2011). Allelic effects were evaluated in comparison to the “null allele” (missing data plus rare alleles) for each associated locus (Breseghello and Sorrells 2006). The large-effect alleles were confirmed with the final plant heights of population 2 by analysis of variance.

## Results

### Phenotypic variation

The mean unconditional plant height (S4) at the flowering stage in Population 1 was 82.4 cm, ranging from 60.0 to 118.0 cm, with a coefficient of variation (CV) of 16.0 %. The CVs of conditional plant height at the three growth periods (PH_1_, PH_2_ and PH_3_) were 9.2, 6.8 and 10.2 %, respectively (Supplementary Table S5). The high values of CV at various growth periods indicated wide phenotypic variation among accessions, which was suitable for association analysis. Compared to PH_1_ and PH_2_, the higher CV of PH_3_ showed that the third growth period (${\text{S}}_{4} |{\text{S}}_{3}$) was more important for plant height development, leading to the obvious differences in plant height in Population 1. The phenotypic data statistics for plant height in Population 2 in eight environments at maturity are presented in Supplementary Table S6.

### Allelic diversity and population structure

A total of 422 alleles were detected by 58 SSR markers in Population 1. The numbers of alleles per locus varied from 3 to 16, and PIC ranged from 0.033 to 0.818, with averages of 7 and 0.519, respectively. A key issue for association mapping is estimation of population structure, which can result in spurious associations between phenotypes and markers. In previous research, about 60 traits were measured at up to 10 environments in the diverse maize germplasm set of 302 inbred lines. Overall, population structure accounts for an average of 9.3 % of the phenotypic variation (Flint-Garcia et al. 2005). The Q-matrix from STRUCTURE can help to reduce the risk of false positives arising from population structure (Bradbury et al. 2007). Twenty-nine whole-genome SSR markers were selected to estimate the population structure of Population 1. The average ln*P*(*D*) value for each *K* (from 2 to 9) is visualized in Supplementary Fig. S1a and the inflection point appeared at *K* = 3 (marked with an asterisk). In addition, the second-order likelihood (Δ*K*) was also calculated. We found that the highest Δ*K* value occurred at *K* = 3, and was much less at *K* = 4–8 (Supplementary Fig. S1b). According to ln*P*(*D*) and Δ*K*, Population 1 was classified into three subpopulations, containing 52, 25 and 41 accessions, respectively (Supplementary Fig. S1c).

### Verification of previous QTL for dynamic developmental plant height by association analysis

Candidate SSR markers for plant height, located in large-effect QTL regions, were used for association mapping in Population 1. Nine loci significantly (*P* < 0.001) associated with plant height were detected 13 times (Table 1). Three loci (*Xgwm11*-*1B*, *Xwmc349*-*4B* and *Xcfd23*-*4D*) were identified in three, two and two periods of plant height growth, respectively. The phenotypic variation explained (PVE) ranged from 14.06 to 28.34 % (Table 1). There were one, two, and seven loci significantly associated with plant height in PH_1_, PH_2_ and PH_3_, respectively; three loci were significantly associated with S4. In total, two markers *Xgwm18*-*1B* and *Xgwm11*-*1B* were detected four times, i.e. *Xgwm18*-*1B* was detected in PH_3_, *Xgwm11*-*1B* was significantly associated with PH_2_, PH_3_ and S_4_, and the genetic distance between them was only 0.7 cM in Ta-SSR-2004 (Somers et al. 2004).

**Table 1:** Phenotypic variation explained by SSR loci significantly (*P* < 0.001) associated with plant height in Population 1

Locus	Chromosome	Position (cM)	PIC	PVE (%)			
				PH_1_	PH_2_	PH_3_	S4
*Xgwm498*	1B	31.1	0.1341				
*Xgwm18*	1B	33.6	0.5410			18.06	
*Xgwm11*	1B	34.3	0.4840		23.55	18.24	19.34
*Xbarc168*	2D	46.7	0.5290	14.06			
*Xgwm249*	2D	63.6	0.2331			15.29	
*Xwmc349*	4B	40.6	0.5526		15.55		16.83
*Xbarc109*	4B	45.9	0.6563			23.96	
*Xcfd23*	4D	32.9	0.5065			20.75	25.82
*Xgwm410*	5A	166.7	0.5606			17.68	
*Xgwm213*	7B	68.2	0.8008			28.34	

*PIC* polymorphism information content, *PVE* phenotypic variation explained

Marker *Xbarc168*-*2D* detected in the first period (PH_1_) was responsible for 14.06 % of the variation in plant height. Two loci on chromosomes 1B and 4B were significantly associated with PH_2_, with PVEs of 23.55 and 15.55 %, respectively. Seven loci identified in PH_3_ were distributed on chromosomes 1B, 2D, 4B, 4D, 5A and 7B. Of them, *Xgwm213*-*7B* showed the largest effect, explaining 28.34 % of the phenotypic variation. At the flowering stage, *Xgwm11*-*1B*, *Xwmc349*-*4B* and *Xcfd23*-*4D* were significantly associated with the plant height. Of them, *Xcfd23*-*4D*, accounting for 25.82 % of the total variation, displayed the largest effect on plant height. *Xgwm11*-*1B* was identified in three periods, with PVEs of 23.55, 18.24 and 19.34 %, respectively (Table 1).

### Allelic effects of associated loci verified in multiple environments

Detecting loci associated with dynamic developmental plant height was not the final objective, but was a prerequisite for its application. It is more significant to search for elite alleles, a major advantage of association mapping compared to biparental family-based linkage mapping. Allelic effects were estimated through comparison with the “null allele” for each associated locus (Breseghello and Sorrells 2006), and large-effect alleles and their phenotypic effects are presented in Table 2. The frequencies of all large-effect alleles were higher than 5 %, ranging from 5.08 to 86.44 %. As for effects at the flowering stage, *Xgwm11*-*1B*_*208*_ and *Xwmc349*-*4B*_*101*_ showed the largest negative (−10.7 cm) and positive (15.1 cm) effects on plant height, respectively. Shorter plant height (74.1 cm) was associated with *Xgwm11*-*1B*_*208*_ (13 accessions), whereas 12 accessions with *Xwmc349*-*4B*_*101*_ were clearly taller (97.5 cm) at flowering. As with previous reports, alleles at one locus could have opposite functions at the same stage (Wen et al. 2008). For *Xgwm18*-*1B*, 12 accessions carrying the 194-bp allele (negative effect −2.6 cm in PH_3_) contributed to shorter plants (77.4 cm), whereas nine accessions with the 189-bp allele (positive effect 9.5 cm) contributed to taller plants (89.5 cm). At flowering, 55 accessions carrying the allele *Xcfd23*-*4D*_*202*_ with a negative effect (−4.2 cm) produced shorter plants (78.1 cm), and 50 accessions with a positive effect (5.1 cm) from the 205-bp allele were taller (87.5 cm).

**Table 2:** Phenotypic effects of marker alleles at loci significantly associated with plant height development in Population 1

Locus	Allele size (bp)	Frequency of allele (%)	Allele effect (cm)			
			PH_1_	PH_2_	PH_3_	S4
*Xgwm498*-*1B*	161	92.37				
*Xgwm18*-*1B*	185	10.17			6.1	
	187	63.56				
	189	8.47			9.5	
	194	10.17			−2.6	
*Xgwm11*-*1B*	197	68.64		−3.8	−2.4	−1.7
	199	5.93		−5.8	1.4	−1.7
	208	11.02		−6.0	−6.5	−10.7
*Xbarc168*-*2D*	172	14.41	−7.0			
	181	24.58	−3.5			
*Xgwm249*-*2D*	167	7.63			9.3	
	182	86.44			−5.0	
*Xwmc349*-*4B*	101	10.17		2.6		15.1
	103	14.41		−1.4		−3.8
*Xbarc109*-*4B*	219	45.76			−2.7	
	234	28.81			5.1	
*Xcfd23*-*4D*	199	10.17			−0.9	−0.1
	202	46.61			0.1	−4.2
	205	42.37			0.3	5.1
*Xgwm410*-*5A*	349	5.08			−3.7	
	352	52.54			−3.3	
	354	5.08			4.3	
*Xgwm213*-*7B*	165	35.59			−4.7	
	169	14.41			−4.7	
	173	13.56			−6.1	
	183	7.63			7.9	

**Fig. 1:**
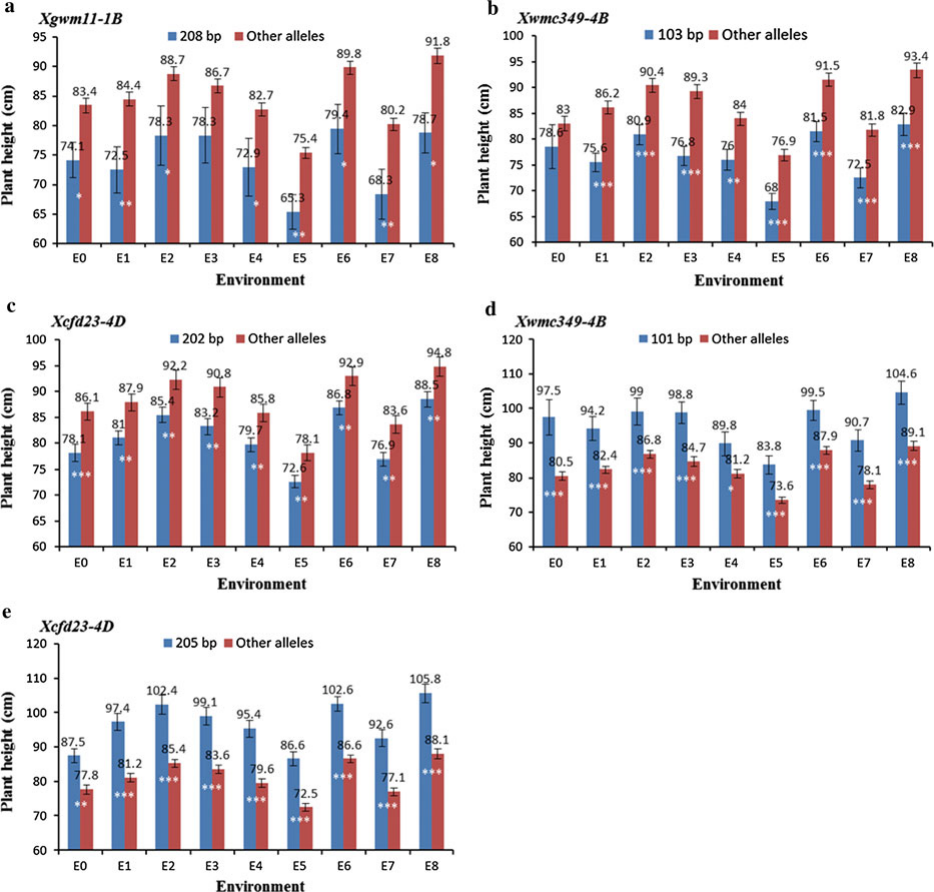
Verification of phenotypic effects of large-effect alleles in two populations in nine environments. Population 1 was planted in E0; Population 2 was planted in E1–E8. *Bars* indicate 2 standard errors. *, **, ***Significant at *P* = 0.05, 0.01 and 0.001, respectively

Population and environment are also critical factors influencing the results of association analysis. In molecular breeding programs, breeders require elite marker alleles associated with target traits that can be repeatedly verified across genetic backgrounds under different environmental conditions. In Population 1, *Xgwm11*-*1B*_*208*_, *Xwmc349*-*4B*_*103*_ and *Xcfd23*-*4D*_*202*_ were detected as having obvious negative effects; *Xwmc349*-*4B*_*101*_ and *Xcfd23*-*4D*_*205*_ showed positive effects at flowering. We then used Population 2 to confirm the large effect alleles of the associated loci in eight environments. The average final plant heights of accessions carrying the large effect alleles (with obvious negative or positive effects) were significantly (*P* < 0.05) or highly significantly (*P* < 0.01, *P* < 0.001) shorter or taller than those without the large-effect alleles in almost all environments (Fig. 1).

### Efficacy of pyramiding large-effect alleles of associated loci

**Fig. 2:**
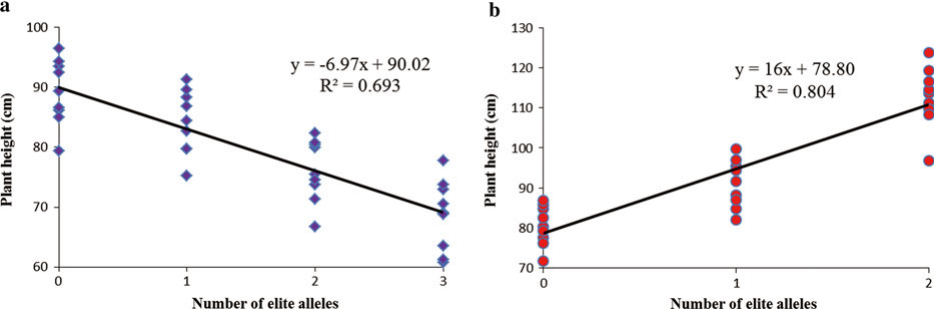
Linear regressions of final plant heights in Populations 1 (118 accessions) and 2 (262 accessions) in nine environments (E0–E8). **a** Plant height and number of alleles with negative effects on height; **b** plant height and number of alleles with positive effects on height

The objective of gene pyramiding in molecular breeding is to combine a series of target alleles in a specific line or variety (Servin et al. 2004). We examined the efficacy of pyramiding multiple alleles with large unidirectional effects in one individual. Three alleles, *Xgwm11*-*1B*_*208*_, *Xwmc349*-*4B*_*103*_ and *Xcfd23*-*4D*_*202*_, with negative effects, and two alleles, *Xwmc349*-*4B*_*101*_ and *Xcfd23*-*4D*_*205*_, exhibiting positive effects on plant height, were identified at the flowering stage. The plant height of genotypes with the three “pyramided” negative alleles was 72.8 cm in Population 1 in E0, and 60.8–77.7 cm in Population 2 in E1–E8. In contrast, the plant height of accessions carrying the two positive alleles was 113.6 cm in Population 1 in E0, and 96.8–123.7 cm in Population 2 in E1–E8 (Table 3). Consistent results indicated that pyramiding of elite alleles associated with plant height produced significantly shorter or taller plants. The linear correlations were highly significant (Fig. 2).

**Table 3:** Phenotypes of accessions with pyramided large-effect alleles associated with final plant height; left columns reduced plant height, right columns increased plant height

Environment	Elite allele	Mean ± SE (cm)	Frequency (%)	Elite allele	Mean ± SE (cm)	Frequency (%)
E0	3	72.8 ± 2.9(A)	8.1	2	113.6 ± 2.3(A)	4.3
	2	82.4 ± 8.1(AB)	4.5	1	84.8 ± 1.5(B)	44.4
	1	79.7 ± 1.9(AB)	40.5	0	77.6 ± 1.5(B)	51.3
	0	86.1 ± 1.6(B)	46.8			
E1	3	69.0 ± 2.3(A)	1.6	2	111.2 ± 2.6(A)	3.1
	2	73.7 ± 1.9(AB)	18.7	1	91.6 ± 2.1(B)	21.5
	1	84.4 ± 1.5(BC)	47.3	0	80.4 ± 1.1(C)	75.4
	0	89.3 ± 1.8(C)	32.4			
E2	3	68.8 ± 3.4(A)	1.6	2	119.2 ± 2.7(A)	3.1
	2	79.9 ± 2.1(AB)	18.4	1	95.5 ± 2.5(B)	21.1
	1	88.3 ± 1.6(B)	47.7	0	84.8 ± 1.2(C)	75.8
	0	93.5 ± 2.0(B)	32.4			
E3	3	70.5 ± 2.4(A)	1.7	2	114.5 ± 2.4(A)	3.3
	2	75.5 ± 2.1(AB)	18.2	1	94.4 ± 2.1(B)	22.3
	1	86.8 ± 1.7(BC)	47.1	0	82.6 ± 1.3(C)	74.4
	0	92.4 ± 2.0(C)	33.1			
E4	3	63.5 ± 2.7(A)	1.6	2	109.7 ± 1.8(A)	3.1
	2	74.6 ± 2.1(AB)	18.3	1	88.3 ± 2.2(B)	21.4
	1	82.7 ± 1.6(B)	47.5	0	79.3 ± 1.2(B)	75.5
	0	86.6 ± 1.9(B)	32.7			
E5	3	60.8 ± 1.6(A)	1.5	2	96.8 ± 2.4(A)	3.1
	2	66.7 ± 1.5(AB)	18.5	1	82.1 ± 1.6(B)	21.6
	1	75.2 ± 1.3(BC)	47.1	0	71.8 ± 0.9(C)	75.3
	0	79.4 ± 1.5(C)	32.8			
E6	3	73.7 ± 3.0(A)	1.6	2	116.5 ± 2.5(A)	3.1
	2	80.4 ± 2.1(AB)	18.3	1	97 ± 1.8(B)	21.8
	1	89.6 ± 1.5(BC)	47.1	0	85.8 ± 1.2(C)	75.1
	0	94.3 ± 1.8(C)	33.1			
E7	3	61.2 ± 1.6(A)	1.5	2	108.3 ± 2.8(A)	3.1
	2	71.3 ± 1.9(AB)	18.5	1	87.1 ± 1.9(B)	21.54
	1	79.7 ± 1.5(BC)	47.3	0	76.3 ± 1.1(C)	75.4
	0	85.0 ± 1.8(C)	32.7			
E8	3	77.7 ± 2.5(A)	1.7	2	123.7 ± 1.6(A)	3.5
	2	80.7 ± 2.3(A)	16.1	1	99.7 ± 2.2(B)	23
	1	91.3 ± 1.8(AB)	47.4	0	86.9 ± 1.3(C)	73.5
	0	96.5 ± 2.1(B)	34.8			

Different letters (in parentheses) in the same column indicate significant differences at *P* = 0.05

Population 1 was planted in E0; Population 2 was planted in E1–E8

## Discussion

### The developmental genetics of plant height

Plant height is controlled by both Mendelian and quantitative genetics (Wang et al. 2010). In this research unconditional and conditional association mapping were employed to dissect the developmental genetics of plant height. Previous studies inferred that alleles at the same genomic location could have opposite genetic effects at different growth stages (Yan et al. 1998b). We obtained similar results. For example, shorter plant height was associated with *Xgwm11*-*1B*_*199*_ (negative effect: −5.8 cm) in the second period (${\text{S}}_{3} |{\text{S}}_{2}$, PH_2_), but in contrast the effect of the same allele was 1.4 cm in the third period (${\text{S}}_{4} |{\text{S}}_{3}$, PH_3_). This further indicated that (1) gene expression occurs selectively (even with opposite effects) at different developmental stages; and (2) several genes with opposite genetic effects may express simultaneously in the same or adjacent genomic regions. However, unconditional analysis considering only final plant height in this research perhaps unavoidably excluded some loci associated with plant height development. This may be one reason why ten loci were detected by conditional analysis, whereas only three loci were detected for final plant height (Table 1).

There were one, two and seven loci significantly associated with PH_1_, PH_2_ and PH_3_, respectively. The participation of different numbers of loci revealed that plant height was a typical quantitative trait, and different genetic systems were responsible for plant height development during ontogeny (Wu et al. 2010). Moreover, seven loci were identified in the third period (${\text{S}}_{4} |{\text{S}}_{3}$, PH_3_), much more than the number of loci found associated with PH_1_ and PH_2_, showing that genes controlling plant height were actively expressed in the third period. Thus, final plant height is the accumulation of genetic effects that occur during the whole growth period, and conditional analysis detects the net effects of gene expression at specific stages.

### Verification of previous QTL and fine mapping

Family-based linkage analysis and association mapping have been widely used in research on quantitative traits. Association mapping based on linkage disequilibrium (LD) can provide high resolution that may contribute to the full use of a potentially large range of allelic variation in natural populations (Remington et al. 2001). Both association mapping and linkage analysis are required to avoid detection of false-positive loci. Therefore, linkage analysis can be used for genomic scans, and association mapping can verify candidate QTL regions and fine mapping. In our research, SSR markers located at candidate QTL regions with large effects in the Hanxuan 10 × Lumai 14 DH population (Wu et al. 2010) were selected for association analysis. Nine loci significantly associated with plant height were detected 13 times (Table 1). The results showed a consistency between linkage analysis and association mapping, and also displayed the fine mapping of QTL through combined linkage and association analysis. For example, an additive plant height QTL, *QPh.cgb*-*1B.1*, was detected in the DH population. The genetic distance between the flanking markers (*Xgwm582*–*Xgwm273*) was 3.9 cM in Ta-SSR-2004 (Somers et al. 2004). Both *Xgwm18*-*1B* (0.2 cM from *Xgwm273*) and *Xgwm11*-*1B*, in the same region, were detected by association mapping in our research, and the genetic distance between them was only 0.7 cM. Some other additive QTL were mapped at known *Rht* loci, such as *QPh.cgb*-*2D.1* which maps to *Rht8*, *QPh.cgb*-*4D.1* to *Rht2* or *Rht10*, and *QPh.cgb*-*5A.6* to *Rht12* (Wu et al. 2010). By association mapping, *Xbarc168*-*2D* and *Xgwm249*-*2D* (in *QPh.cgb*-*2D.1*), *Xcfd23*-*4D* (1.6 cM near to one flanking marker of *QPh.cgb*-*4D.1*), and *Xgwm410*-*5A* (one of the flanking markers of *QPh.cgb*-*5A.6*) were detected in our research. *Xwmc349*-*4B* is closely linked with *Rht*-*B1* and *Xgwm410*-*5A* is linked with *Rht12* at a distance of 11.0 cM (Ellis et al. 2005; Korzun et al. 1997; Somers et al. 2004).

Flint-Garcia et al. (2003) proposed that if a marker is too close to the causal gene, the frequency of the major allele is so high that other alleles become null alleles (low frequencies); the very low diversity of the marker will then fail to be detected by association mapping (Flint-Garcia et al. 2003). The genetic locations of *Xgwm498*-*1B*, *Xgwm18*-*1B* and *Xgwm11*-*1B* were 31.1, 33.6 and 34.3 cM, respectively, in the consensus map Ta-SSR-2004 (Somers et al. 2004). *Xgwm18*-*1B* and *Xgwm11*-*1B* were detected in our research, but it was surprising that *Xgwm498*-*1B* was not detected. The frequencies of the major alleles of *Xgwm18*-*1B* and *Xgwm11*-*1B* were 63.56 and 68.64 %; the PIC values were 0.5410 and 0.4840, respectively. The frequency of the major alleles of *Xgwm498*-*1B* was 92.37 %, and the PIC was only 0.1341 (Tables 1, 2). Thus, it is inferred that if a causal gene for plant height was near the genomic region of *Xgwm498*-*1B*, it should have been selected in breeding. Because of the effect of “selection valleys” (close proximity to the selected gene, and consequently low genetic diversity at the locus) (Barrero et al. 2011), *Xgwm498*-*1B* was at the bottom of the valley, and failed to be detected. *Xgwm18*-*1B* and *Xgwm11*-*1B*, on the other hand, flanked the valley, and were thus detected in our research. The same “selection valleys” in this chromosome region were reported to associate with plant height (Barrero et al. 2011).

### Stable molecular markers and enough elite alleles determine the future of gene pyramiding

New strategies of MAS are flourishing in the genomics era, and the explosive growth in the number of QTL reminds us to ask (1) how many previously reported QTL could be repeatedly detected or verified, and (2) are there enough elite alleles in germplasm resources? It has been proposed that the narrow genetic base of modern crop cultivars is a major problem for further improvement of crop productivity (Abdurakhmonov and Abdukarimov 2008). Genetic diversity in germplasm resources provides a good way to reduce the problem of the high degree of similarity among cultivars. It is therefore important to search for elite alleles at loci associated with important traits. Different alleles of a causal gene can lead to drastic phenotypic differences; for example, a single SNP caused loss of seed shattering during rice domestication (Konishi et al. 2006). Therefore, exploration of elite alleles is a critical region for germplasm managers and plant breeders looking for alleles of interest in germplasm collections rather than as sequences in GenBank (Famoso et al. 2011). Many studies show that marker-based strategies of pyramiding are effective (Sacco et al. 2013; Werner et al. 2005). However, an essential prerequisite for gene pyramiding is stable molecular markers (Brar et al. 2000); that is, the elite alleles of associated loci must be repeatedly detected or verified without being limited by interactions with genetic background or environment. In our research, five large-effect alleles with obvious negative or positive effects were identified in Population 1 in E0 and validated using Population 2 in eight environments (E1–E8) (Table 2; Fig. 1). We also observed that genetic background has a great influence on the effect of elite alleles. For example, varieties Jimai 32 and Jinguang carried the negative allele (*Xwmc349*-*4B*_*103*_), but showed extremely tall final plant heights (118 cm and 108 cm) compared to the average of all accessions with *Xwmc349*-*4B*_*103*_ in Population 1 in E0 (data not provided). Rare individuals like Jimai 32 and Jinguang can hide the effect of *Xwmc349*-*4B*_*103*_ in small populations, but cannot hide the effect in large populations. Therefore, it is easy for us to understand why no significant difference in plant height was detected between accessions carrying *Xwmc349*-*4B*_*103*_ and others in E0 in Population 1 (118 accessions), but there was extreme significant difference across E1–E8 in Population 2 (262 accessions, Fig. 1). Pyramiding elite alleles associated with plant height showed that the greater the number of elite alleles, the higher (or lower) the resulting plant height (Table 3; Fig. 2). The obvious dosage effect shows that pyramiding elite alleles for a target trait has great potential for wheat breeding.

Many important traits are controlled by QTL. The combination of linkage analysis and association mapping provides an efficient method of finding elite alleles in natural populations. But as QTL are based on statistical calculations, verification is needed to confirm the effectiveness of such alleles (Ashikari and Matsuoka 2006). In conclusion, stable molecular markers and a sufficient number of elite alleles will determine whether the pyramiding of elite alleles by MAS will or will not be effective in plant breeding.

## Electronic supplementary material

Below is the link to the electronic supplementary material.

Supplementary Table S1 Backcross introgression lines (BILs) in Population 1 (XLS 30 kb)

Supplementary Table S2 262 accessions of Population 2 and their origins (XLS 53 kb)

Supplementary Table S3 Candidate SSR markers for association mapping in Population 1 (XLS 26 kb)

Supplementary Table S4 SSR markers for evaluating population structure of Population 1 (XLS 26 kb)

Supplementary Table S5 Conditional phenotypic values for plant height of Population 1 during three growth periods and unconditional plant height at flowering (XLS 24 kb)

Supplementary Table S6 Unconditional plant height of 262 accessions at maturity stage (XLS 24 kb)

Supplementary Fig. S1 Population structure of Population 1 based on 29 genome-wide SSR markers. a, b: Population structure as determined by ln*P*(*D*) and Δ*K* over five repeats of STRUCTURE analysis; c: Structure analysis revealed three sub-populations. Each accession is represented by a vertical bar, and the colored segments within each bar reveal the proportion of each subpopulation (PPT 859 kb)
