# Supplementary figures and images for: Efficacy of pyramiding elite alleles for dynamic development of plant height in common wheat
Source: Mol Breed. 2013 Jun 6;32(2):327–38. doi: 10.1007/s11032-013-9873-5 (PMC3748324; doi:10.1007/s11032-013-9873-5)

## Slide 1
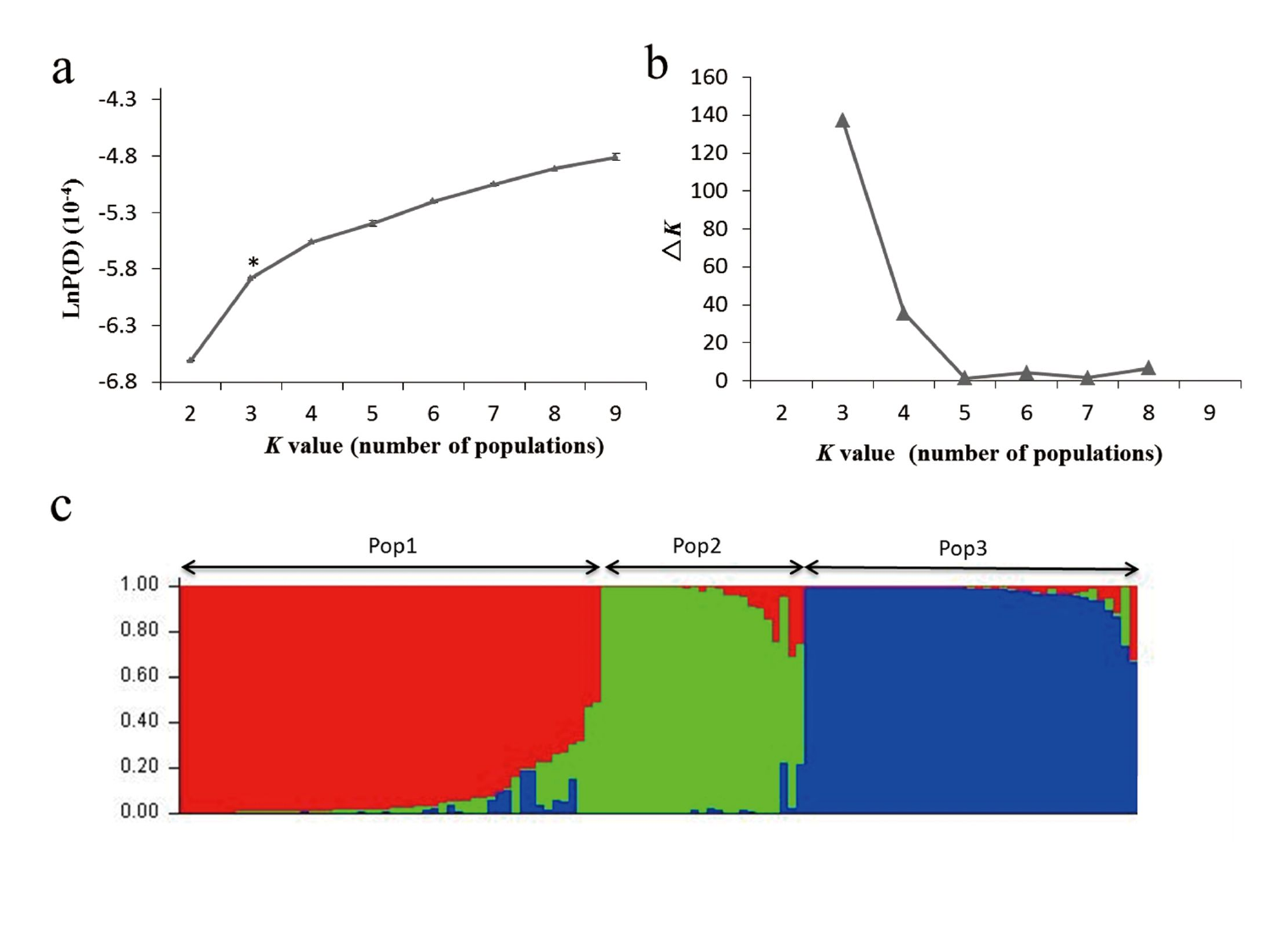

Supplement: Supplementary file 7 — Population structure of Population 1 based on 29 genome-wide SSR markers. a, b: Population structure as determined by lnP(D) and ΔK over five repeats of STRUCTURE analysis; c: Structure analysis revealed three sub-populations. Each accession is represented by a vertical bar, and the colored segments within each bar reveal the proportion of each subpopulation (PPT 859 kb) [file 11032_2013_9873_MOESM7_ESM.ppt]
